# Advances in Resistance Welding of Fiber-Reinforced Thermoplastics

**DOI:** 10.3390/ma17194693

**Published:** 2024-09-24

**Authors:** Zhanyi Geng, Shibao Yu, Shiyuan Wang, Zengtai Tian, Zhonglin Gao, Kaifeng Wang, Yang Li

**Affiliations:** 1 School of Materials Science and Engineering, Tianjin University, Tianjin 300354, China; 2 Tianjin 707Hi-Tech Co., Ltd., Tianjin 300402, China; 3 Key Laboratory of Mechanism Theory and Equipment Design of Ministry of Education, Tianjin University, Tianjin 300354, China; 4 International Institute for Innovative Design and Intelligent Manufacturing Tianjin University in Zhejiang, Shaoxing 312000, China

**Keywords:** fiber-reinforced thermoplastic (FRTP), resistance welding, heating element, joint performance, weld quality, numerical simulation, thermoset composite

## Abstract

Fiber-reinforced thermoplastics (FRTPs) have become a new generation of lightweight materials due to their superior mechanical properties, good weldability and environmental resistance, potential for recycling, etc. The market for FRTPs is expected to grow at a compound annual growth rate (CAGR) of 7.8% from 2022 to 2030. Many researchers have been trying to solve the problems in their processing and joining process, and gradually expand their application. Resistance welding is one of the most suitable techniques to join FRTPs. This paper summarizes the research progress of FRTP resistance welding in terms of the basic process of FRTP resistance welding, factors affecting joint performance, joint failure behavior, numerical simulation, weld quality control, and resistance welding of thermoplastic/thermoset composites. The objective of this paper is to provide a deeper insight into the knowledge of FRTP resistance welding and provide reference for the further development and application of FRTP resistance welding.

## 1. Introduction

**Figure 1:**
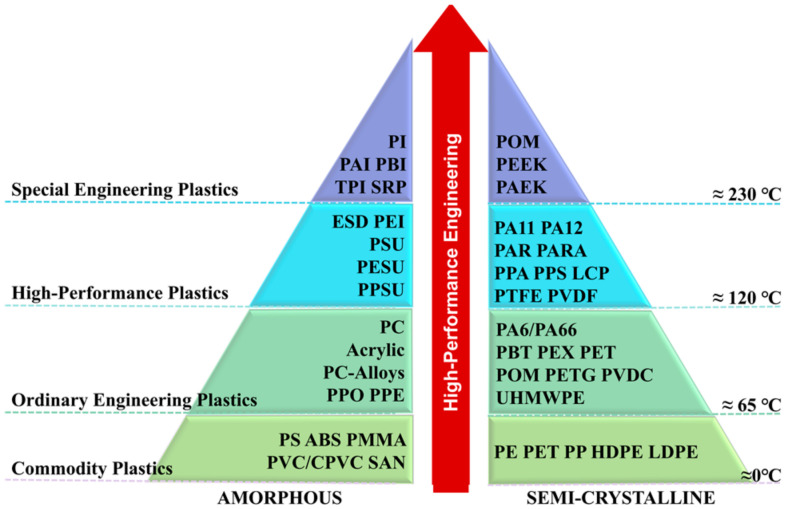
Thermoplastic resins with different properties [26].

Lightweight design is the common pursuit in the fields of automobile manufacturing, rail transportation, and aerospace to achieve energy savings and emission reductions [1]. With polyether ether ketone (PEEK), polyphenylene sulfide (PPS), polyetherimide (PEI), polyamide (PA), polyimide (PI), polysulfone (PSU/PSF), polypropylene (PP), and other thermoplastic resins (Figure 1) as the matrix [2], and carbon fiber (CF), glass fiber (GF), aramid fiber (AF), and so on as the reinforcing phase, fiber-reinforced thermoplastic composites (FRTPs) have the following advantages:

(a) Excellent mechanical properties: high specific strength, specific stiffness, specific modulus, excellent toughness, fatigue resistance, impact resistance, and fracture toughness [3];
(b) High productivity: easy to assemble, fast molding and processing, short production cycle, easy to realize automated production [4];
(c) Environmental resistance: storage without environmental and time requirements, high resistance to halogenated hydrocarbons, refrigerants and oils and greases, good resistance to high temperatures and corrosion, good flame retardancy [5,6];
(d) Fluid resistance and abrasion resistance: low permeability and hygroscopicity, high tolerance to fluids such as seawater, solvents, transmission fluids, etc., and low friction factor [2,7];
(e) Good weldability: repeatable molding, no solidification reaction during melt–solidification [8];
(f) Repairable: polymer chains diffuse and entangle with each other to form new cross-linking structures during repeated molding, and the performance is not affected by heating and the number of times of molding [9,10];
(g) Recyclable: it is easy to be recycled and has a high recycling rate, which is green and environmentally friendly [11,12].

Therefore, FRTPs gradually break the monopoly of traditional thermoset composites (TSCs) [13] and become a favored new generation of lightweight materials and key strategic materials supporting the new generation of high-tech fields such as aviation and aerospace [14,15], the manufacturing industry [16,17], energy power [18], transportation [19,20], defense industry [21], construction [22], and household goods and electronic products [22]. Taking the aviation and aerospace field as an example, the application proportion of composite materials on large passenger aircrafts has gradually increased, and FRTPs have been gradually applied to aircraft skins, ailerons, fairings, beam ribs, elevator rudders, structural panels, and other components [23,24,25,26].

In addition, the manufacturing of FRTPs has gradually tended to be low-cost, high-performance, high-precision, high-efficiency, automated, and intelligent, and its components have shown the trend of large-scale, integrated, and monolithic [27]. However, the high viscosity of the thermoplastic resin of FRTPs and the internal reinforcing fibers increase the difficulty in manufacturing FRTP components [28]. Therefore, efficient and reliable joining technology is crucial to fully utilize the excellence of FRTPs [29,30]. Traditional mechanical joining has a straightforward process, but the processing time is long and labor-intensive, and the drilling of holes also causes problems such as stress concentration, damage to matrix fibers, and weight gain [31,32]. Adhesive bonding effectively avoids the above shortcomings, and the joint fatigue resistance is superior, but the adhesive storage life is limited, and there are high surface quality requirements, a long curing time, low peel strength, sensitivity to the environment and pollution, and it is not easy to bond chemically inert FRTPs [33]. Welding not only improves efficiency, reduces the cost, and reduces the workload of assembly, but also avoids stress concentration and fiber breakage caused by drilling [28], which is an obvious advantage when joining FRTPs.

The main welding methods for FRTPs include electromagnetic welding (resistance welding [4], induction welding [34,35], etc.), friction welding (ultrasonic welding [36,37], vibratory welding [38], etc.), and thermal welding (laser welding [39], infrared welding [40], microwave welding [41], hot air welding [42], hot plate welding [43], etc.). Among these methods, resistance welding has the features of low cost, high efficiency, easy for automation, suitable for thick sheets, and so on [44,45,46]. It has been widely used in aircraft manufacturing. For example, Fokker Aircraft Structures [47,48] used resistance welding to successively attach the main landing gear doors of the Fokker 50, the J-nose of the Airbus A340-600, and the skin and side panels of the leading edge of the wing of the Airbus A380. In 2018, Premium Aerotec GmbH [49], in Germany, realized the first resistance welding assembly of the pressure bulkhead of the Airbus A320 and developed an automated equipment. The European Union [50,51] developed a resistance welding technique to manufacture the first and the largest thermoplastic fuselage demonstrator under the Multifunctional Fuselage Demonstrator (MFFD) project [52]. The German Aerospace Center (DLR) installed fuselage transverse reinforcement by resistance welding [53], which significantly reduced the production time and cost.

With the increasing demand for resistance welding technology in a composite structure assembly, a large number of studies on FRTP resistance welding have emerged in recent years. However, a systematic review on FRTP resistance welding is very limited [4,32]. This paper analyzes the basic process of FRTP resistance welding, factors affecting joint performance, failure analysis, numerical simulation, process control and quality inspection, and resistance welding of thermoplastic/thermoset composites. The challenges and latest research progress of FRTP resistance welding are summarized and discussed, with the aim of providing a reference for the development and application of FRTP resistance welding.

## 2. Basic Process in FRTP Resistance Welding

### 2.1. Heat Production Mechanisms

**Figure 2:**
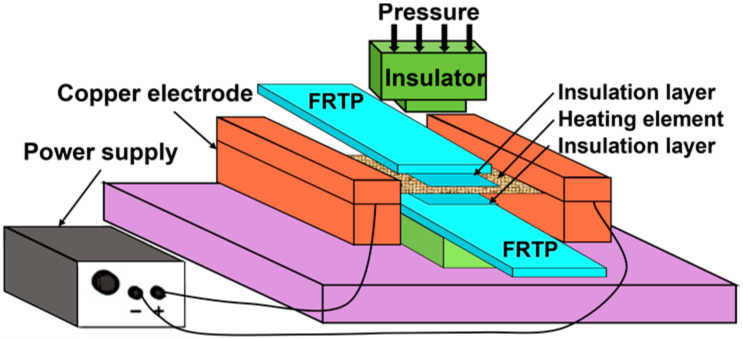
Schematic of FRTP resistance welding [56].

Figure 2 shows the schematic of FRTP resistance welding. Since FRTPs are not conductive, their resistance welding requires placing a heating element (HE) at the interface between the workpieces. The heating element is usually stainless steel (SS) mesh or carbon fiber material. When the current flows through the HE, the generated resistance heat provides heat for the entire welding process. The generated resistance heat follows the Joule ‘s law as described in Equation (1):

(1) $$Q=I^{2}Rt\;[J]$$

where *Q* is the resistance heat generated, *I* is the welding current, *R* is the resistance of the HE, and *t* is the welding time. When the interface temperature reaches the glass transition temperature T_g_ (amorphous polymer) or the melting point T_m_ (semi-crystalline polymer) of the FRTP matrix [10,54,55], the molecular mobility of the polymer chains increases, and the thermoplastic matrix begins to melt. Under the action of a certain welding force, the two FRTP workpieces are in close contact, and the polymer chains can diffuse and entangle with each other, forming a reliable joint after pressurized cooling [30,56,57,58].

### 2.2. Process of Joint Formation

**Figure 3:**
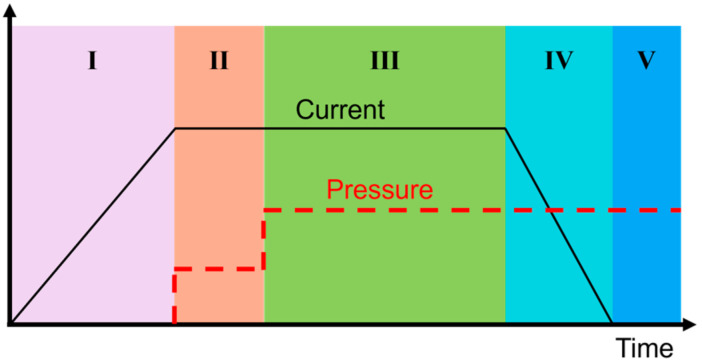
FRTP resistance welding control process [59].

Lu et al. [59] divided the resistance welding process into the following five stages according to the temperature, current, and pressure changes (Figure 3):

I: The welding current increases at a rate and Joule heat generates in the heating element to heat the workpieces, which expand and squeeze against the heating element, stopping the increase in the current when the interface temperature reaches the melting point T_m_ (for semi-crystalline polymers) or the glass transition temperature T_g_ (for amorphous polymers);

II: Keep the current unchanged and apply the initial pressure (P_s_) to eliminate the gap between the heating element and the workpieces, while ensuring that the interface between the workpieces is heated evenly;

III: Keep the current constant and continue to increase the welding pressure (P_m_); the molten resin matrix flows under pressure and the molecular chains diffuse and entangle with each other;

IV: Reduce the current at a certain rate, the workpiece cools and solidifies under pressure (P_m_), and the molten polymer stops flowing and begins to solidify;

V: Cut off the welding current, the joint and the entire welding system continue to cool and shrink under pressure, completing the welding.

### 2.3. Edge Effect

As early as 1988, Eveno et al. [60] found that due to the poor natural heat transfer between the HE and the air, the HE temperature when exposed to the air is higher, and the temperature at the edge of the workpieces is significantly higher than that in the interior of the workpieces, which is usually referred to as the “edge effect”. The high edge temperature in turn causes the resin matrix at the edge part along the current direction to melt first, and then gradually spread to the center part and the side (Figure 8 of reference [61]), leading to overheating at the edge region, excessive surface melting, large joint deformation, and reduced joint strength. Methods such as applying pulse voltage or linear voltage [62], changing the clamping distance [63], increasing the power appropriately [63], cooling the edge of the workpieces [64], and reducing the length of the heating element in the joint area [65] can effectively attenuate or eliminate the edge effect and guarantee the joint performance.

Among these methods, optimizing the clamping distance is expected to be the simplest and most effective way to improve the temperature uniformity in the welding interface, and therefore expand the process window [66]. Dube et al. [67] found that a reduction in the clamping distance to 1.5 mm effectively improved the temperature uniformity in the resistance welding of CF/PEEK. Talbot et al. [68] found that the clamping distance and the heat conduction along the length of the base material affect the interfacial temperature uniformity, and an optimized clamping distance can also effectively expand the process window. Brassard et al. [69] found that the edge effect produced by the excessive distance resulted in the edge being 10 °C higher than the central region when the clamping distance was 1.5 mm. They then [70] found that the optimum clamping distance for CF/PEEK resistance welding was approximately linearly correlated with the power density and consistently differed by 0.4 mm from the clamping distance at which the edge began to thermally degrade.

### 2.4. Shunting Effect

**Figure 4:**
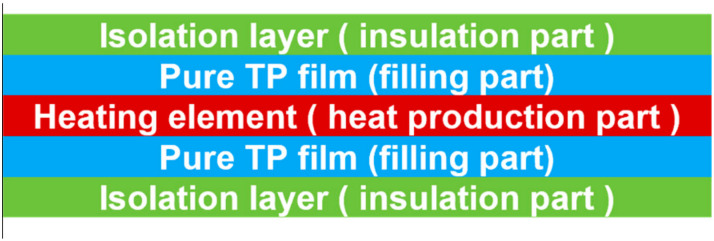
Typical structure and composition of a resistance welding interlayer [72].

In addition to the edge effect, the contact between the HE and the conductive fibers inside the workpieces will generate new conductive paths, leading to current shunting (also known as current leakage), which reduces the HE current density [60] and affects the uniformity of the interface temperature distribution (Figure 7 of reference [60]) [62,71]. To avoid shunting during welding, the HE should be insulated from any conductive part at the interface, so an insulating layer is often added on both sides of the HE [4], and a pure resin film identical to the matrix of the composite material is chosen as the transition layer (Figure 4), which promotes a better flow of the polymer chain of the matrix to diffuse and fill the interfacial gap [32]. Due to the excellent insulating property of the resin, many researchers also directly choose the same pure resin film for the base material as the insulating layer.

## 3. Factors Affecting the Performance of Resistance-Welded Joints

The resistance-welded joint performance is affected by process parameters such as the welding power (welding current [73]), welding time, and welding pressure, as well as the material properties of the HE. In addition, the fiber size and orientation, moisture and oil, and ultrasonic assistance all affect the final joint properties.

### 3.1. Welding Process Parameters

**Figure 5:**
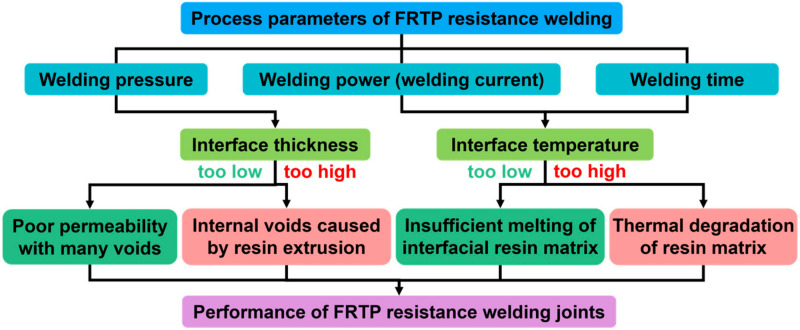
Effect of FRTP resistance welding process parameters on joint properties [55].

Figure 5 shows the effect of the FRTP resistance welding process parameters on the joint properties. When the welding input energy (current or time) is too small, the heat generation is insufficient, leading to insufficient melting of the interface resin. If the heat input is too large, the interface temperature rises rapidly, and the resin will be degraded due to overheating. When the welding pressure is too small, there are more voids caused by uncompacted fibers [74]; when it is too large, more resin will be extruded at the interface, reducing the interfacial connection strength.

To determine the influence of each process parameter, many researchers adopted the Taguchi method, ANOVA, response surface method, orthogonal method, or other research methods, all of which concluded that the welding current (power) has the greatest influence on the joint performance, as listed in Table 1. Du et al. [56] plotted the process window of GF/PP resistance welding and established the quantitative relationship between the three-point bending strength and process parameters, and the error with the experimental value was only 6.4%. Hou et al. [75] used the response surface method to plot the process window of GF/PP resistance welding, and Lu et al. [76] plotted the process window of CF/PPS resistance welding.

It should be noted that the selection of welding parameters is affected by the nature of the materials to be welded, such as the melting point, thermal conductivity, viscosity, crystallization behavior, and so on [55,73,81].

#### 3.1.1. Welding Power and Energy Input

Welding power usually refers to the heating element power to produce Joule heat after energization, generally expressed in terms of power per unit area (power density), see Equation (2):

(2) $$P=\frac{I^{2}R}{lb}\left[\frac{W}{m^{2}}\right]$$

where *P* is the power density; *I* is the current flowing through the HE; *R* is the resistance of the HE; *l* is the length of the welding zone; and *b* is the width of the welding zone. Appropriate welding power helps to improve the welding efficiency and joint mechanical properties. Since the heat from the welding interface will conduct to the base material, fixture, and the environment, if the power density is low, the time required to melt the substrate will be very long. Lu et al. [76] found that when the power is low and the time is long, although the heat diffusion inside the joint is sufficient, there are problems such as joint softening and deformation, which also increase the economic cost and are not conducive to practical production. The rapid concentration of heat at the welding interface when the power density is too high can lead to the overheating decomposition of the polymer matrix, insufficient melting due to increased resin mobility, and joint deformation, which reduces the joint performance [4,75,82]. Hou et al. [61] found that with the increase in the power, the maximum LSS increased and then decreased, and the joints had the highest LSS when the power was 140 kW/m^2^.

To improve the interface heating characteristics, researchers have also adopted a ramp voltage (or ramp current, ramp power); that is, the voltage increases from a set value at a constant rate until the interface temperature reaches a predetermined processing temperature and then stops heating. Due to the constant heating rate, the temperature distribution at the welding interface is more uniform. However, the technique requires a higher power supply and also real-time detection of the interface temperature. Georges et al. [83] found that the welding time of pulsed resistance welding is less than 20 s for the rated power of 400~600 kW/m^2^. Yousefpour et al. [84] selected the ramp power and found that the LSS of CF/PEEK joints was the largest at 49.01 MPa (95% confidence level) when the processing temperature was 450 °C and a medium heating rate of 526 °C/min was selected. They [85] then analyzed the input energy of 2800 kJ/m^2^, 3360 kJ/m^2,^ and 3600 kJ/m^2^ for ramp voltage, pulse voltage, and constant voltage, respectively, in conjunction with finite element simulation at a 450 °C processing temperature and 40 s welding time.

#### 3.1.2. Welding Time

Welding time is another important factor affecting the joints. For a constant power input, the total heat is proportional to the welding time. Therefore, a proper welding time is critical to the joint quality. In general, it should be ensured that the temperature of the welding interface is higher than the melting temperature of the thermoplastic matrix and lower than its thermal decomposition temperature. When the welding time is too short, the heat input is insufficient, which results in poor resin flow and weak joint strength. When the welding time is too long, it leads to resin decomposition and joint softening and deformation, which reduces the joint strength. It was found that with the selection of metal mesh [86], carbon fiber [79], or a new heating element [87], with the increase in the welding time, the joint LSSs all showed a tendency of increasing and then decreasing, and gradually tended to stabilize, and were independent of the type of resin film [86], but the joint LSSs under some processes had a platform stage [61].

Yan et al. [88] found that the CF/PEEK joint LSS increased almost linearly with the increase in the welding time (0~10 s). Yu et al. [78] found that with the extension of the welding time, the joint crystallinity increased, which led to the increase in joint brittleness, and the LSS first increased and then decreased, and eventually reached stability. 

#### 3.1.3. Welding Pressure

The welding pressure in the resistance welding of FRTPs can make the joint interface contact closely, eliminate the gap of the interface, and promote the mutual diffusion and entanglement of the polymer chains of the resin matrix to ensure the joint quality. If the welding pressure is large, there will be problems such as resin extrusion, fiber exposure, and severe deformation of the joint. If the welding pressure is small, the porosity at the interface will be high, and the interface bonding and joint performance will be reduced [4,89]. The constant displacement method and constant pressure method are common pressure control methods for FRTP resistance welding.

The constant displacement method needs to set an initial pressure and a constant displacement, which can effectively control the final joint thickness, and the operation is relatively simple. However, the actual pressure increases first and then decreases with the heating, melting, and cooling of the resin, which cannot guarantee the consolidation quality [90,91,92]. Eveno et al. [60] found that because the maximum pressure in the CF/PEEK constant displacement resistance welding was higher than the initial pressure, the molten resin was extensively extruded. During cooling consolidation, ‘dry adhesion’ occurs due to the significant reduction in pressure, which cannot guarantee the joint quality [93]. Howie et al. [94] found that the LSS is inversely proportional to the interface thickness, and the interface thickness is negatively correlated with the temperature and pressure. When the temperature is greater than the T_g_ of the matrix, the joint LSS is the largest. When the welding pressure was greater than 0.43 MPa, the joint LSS appeared as a plateau value. Yan et al. [88] found that when the welding pressure was 0.4 MPa, the LSS of the CF/PEEK joints reached a peak of 18 MPa. When the pressure was too high, the joint was seriously deformed, the molten matrix was extruded too much, and the joint LSS was reduced.

The constant pressure method only needs to set a constant pressure value during the welding process. Because the pressure is always constant, it is more conducive to the diffusion of the polymer chain during welding [95] and the discharge of the interface gas during cooling and consolidation [62]. The joint mechanical properties and stability are better. Therefore, this method has been widely used by researchers, but it is difficult to predict the final joint thickness [96]. Ageorges et al. [96] found that CF/PEI was well welded when the pressure was 0.2~1.6 MPa. When the pressure was about 0.5 MPa, the joint performance was the best. Warren et al. [97] found that there were many porosities at the interface of GF/PET joints when the welding pressure was small (0.0965 MPa), and the porosities disappeared when the pressure increased to 0.345 MPa. When the pressure is 0.689 MPa and 1.379 MPa, the joint LSS is 18.1 MPa and 25.61 MPa, respectively. Shi et al. [74] found that the temperature distribution during welding was uneven, resulting in insufficient pressure at the joint center and more porosities. The pores can be divided into two types: fiber decompression-induced pores generated by residual stress release in the heated base material and residual volatilization-induced pores generated by the residual volatilization of internal solvents or water. The critical pressures for eliminating both types of pores were found to be 0.4 MPa and 1.5 MPa, respectively.

### 3.2. Heating Element

In resistance welding, the HE not only affects the temperature distribution of the interface and the flow of the molten resin, but also affects the joint stress distribution and environmental adaptability [31,93,98,99]. At present, FRTP resistance welding usually uses metal mesh, carbon fiber material, and other new materials as an HE [55]. Therefore, it is important to design or select the appropriate HE to ensure the performance of FRTP resistance-welded joints.

The resistance of the HE is affected by many factors such as the material type, shape, temperature, impurities, and defects [60,99,100,101]. Assume that an HE is isotropic; its resistance can be described by the following Equation (3):

(3) $$R=\rho \frac{l}{b}[\Omega ]$$

where *R* is the resistance of the HE; *l* is the length of the conducting path of the HE; *b* is the width of the conducting part of the HE; and *ρ* is the resistivity of the HE. The resistance of an HE is directly proportional to its length and resistivity and inversely proportional to its width. Eveno et al. [60] and Hou et al. [82] have determined that the resistivity of a CF single-layer HE is about 0.2 Ω·m.

In addition, the resistance of the HE is also related to the temperature. The resistance of an SS metal mesh at 340 °C is about 25% of that at room temperature [100]. For carbon fiber, the temperature has a relatively small effect on its resistance [99]. As the temperature increases, the number of free charge carriers increases, and the conductivity of carbon fiber increases [102]. Researchers have found that the resistance of CF/PEEK HE at 340 °C is 6.3% [60], 10% [98], and 16% [100] lower than that at room temperature.

To further improve the compatibility and bonding between the HE and the resin matrix, researchers have proposed to use surface modifiers (silane coupling agents, surfactants, etc.) to modify the surface of the HE, and to add fillers such as short-fiber materials, nanomaterials, etc., to enhance the bonding strength and mechanical properties of the resin at the interface [78].

#### 3.2.1. Metal Mesh Heating Element

**Figure 6:**
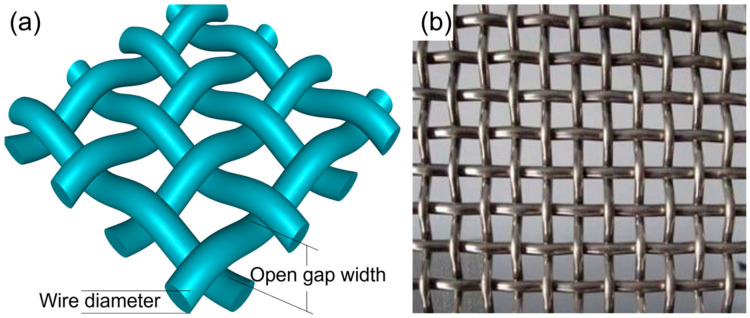
Metal mesh heating element: (**a**) schematic diagram; (**b**) physical drawing [62].

Metal mesh (Figure 6) has been widely used in the resistance welding of FRTPs due to its low cost, good electrical and thermal conductivity, and its more stable and predictable change in resistance with an increasing temperature [10,62]. Researchers have studied the effect of process parameters on properties such as the lap shear strength (LSS) and interfacial shear strength (IFSS) of the joints. They also improved the compatibility of a metal mesh with the resin by adding insulating layers on both sides of the metal mesh to prevent current shunting. Table 2 summarizes the research in the resistance welding of FRTPs when using metal mesh heating elements. It seems that the selection of the HE (dimensions) is rather random. Some researchers investigated the influence of metal mesh HE dimensions on the resistance welding process. For example, Requena et al. [7] compared 25-mesh and 250-mesh SS with 160-mesh bronze mesh and found that 250-mesh SS had the best heating characteristics and a wider range of available sizes. Yu et al. [78] compared 40-mesh, 100-mesh, and 200-mesh brass mesh and selected a 100 μm PPS film as the insulating layer. They found that the heating rate of 100-mesh brass mesh was faster, and the maximum LSS of CF/PPS was 10.73 MPa when the power was 124~128 kW/m^2^ and the welding time was 60 s. Some research demonstrates that a metal mesh HE with a smaller wire diameter and large open gap was beneficial for the flow and expansion of the molten resin, which contributes to a higher joint strength [62,72,103]. However, there is still no standard or guidance on how to select a suitable metal mesh HE for specific materials. An important reason for this suitability is that the commercially available stainless steel meshes do not form series, such as metal meshes with the same wire diameter but different open gaps. In addition, there is also no guidance on how to determine the thickness of the insulating layer. In a word, the trial-and-error method is currently commonly used to study the metal mesh HE, which lacks theoretical guidance.

To further improve the bonding strength between the metal mesh HE and the resin matrix, and then improve the joint performance, researchers have proposed the surface modification of metal mesh. Table 3 summarizes these surface modification methods. However, the above studies have not further investigated the impact of coatings on the joint fatigue performance [71].

#### 3.2.2. Carbon Fiber Heating Element

**Figure 7:**
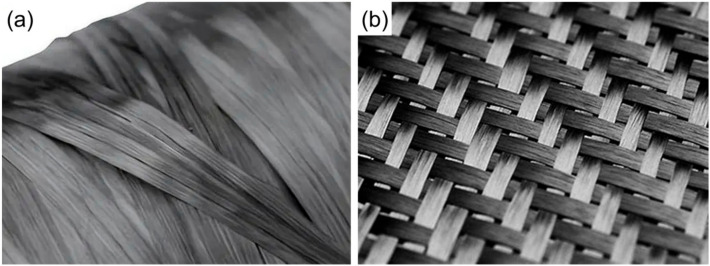
Carbon fiber heating element: (**a**) unidirectional carbon fibers; (**b**) woven carbon fibers.

Compared with metal mesh HEs, carbon fiber HEs have a lower density, and will not increase the weight of the joint [4]. In addition, as one of the reinforcing phases of FRTPs, they have better compatibility with the matrix and can effectively ensure the joint corrosion and fatigue resistance. Therefore, carbon fiber HEs have received extensive attention [59]. Commonly used carbon fiber HEs are mainly unidirectional carbon fibers (Figure 7a) or woven carbon fibers (Figure 7b). Under the same welding conditions and similar fiber volume fraction, the resistance of the woven carbon fibers is greater, and the interface temperature distribution is more uniform, and the welded joints made with woven carbon fiber heating elements are not easy to tear during transverse loading and have a better overall performance [64,75,95,98].

Table 4 summarizes the studies on FRTP resistance welding with carbon fiber HEs. Comparing Table 2 and Table 4, the strength of joints obtained using a metal mesh HE is generally higher than that obtained using a carbon fiber HE. Hou et al. [61,81] found a more uniform interfacial temperature distribution and a wider process window when using an SS mesh/pure PEI film HE compared to using a woven CF/PEI prepreg HE. While the average LSS was essentially the same, the latter joint was chosen to have a better strength uniformity and higher fracture toughness.

Roy et al. [77] found that the CF/PLA joint tensile breaking load was increased by 31% and the compressive breaking load was increased by 63% when using a carbon fiber HE compared to SS mesh. The joint tensile damage load increased by 187% and the compressive damage load by 323% when carbon fiber HEs were used compared to polypyrrole (PPy) conductive materials.

Yao et al. [107] investigated the effect of different HEs on CCF/PEEK resistance welding. It was found that although the structure of SS mesh was chosen to favor resin flow and impregnation with a better process consistency, it was less compatible with the resin. T700 plain fabric was chosen to have excellent compatibility with the matrix, but the tightly woven structure hindered the flow and impregnation of resin, and the joint LSS fluctuated greatly. A single layer of T700 Carbon Fiber Spreader Fabric was selected with less thickness than T700 Plain Fabric, but resin flow caused surface fiber bending.

**Figure 8:**
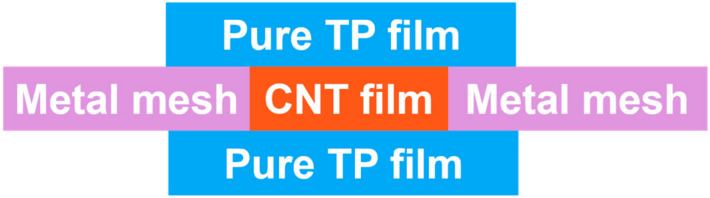
Schematic diagram of the structure of the hybrid HE [133].

To solve the problems of the complicated pretreatment of CF heating elements and easy oxidization and ablation, as well as to further improve the joint strength, researchers have proposed the following methods. Tanaka et al. [132] in situ deposited a CNT on the CF heating element, and the increase in the CF conductive area also reduced the resistance and increased the heat production, which improved the temperature of the welding interface and the joint strength. However, excessive CNTs are not conducive to resin impregnation and reduce the bonding strength. Zhao et al. [87] found that the LSS of GF/PEI joints increased by 23.6% after a CNT was prepared on CF. Subsequently, they [133] designed a CNT film and SS mesh hybrid HE (Figure 8). It was found that the CNT film fragments gradually entered the PEI resin during the welding process, which enhanced the interface bonding. The LSS of CF/PEI joints increased from 2.1 MPa to 14.8 MPa, and the loading time increased from 5 s to 15 s, and the cracks were also significantly reduced.

#### 3.2.3. New Types of Heating Elements

Although metal mesh HEs and carbon fiber HEs have been widely used in the resistance welding of FRTP, they still have some limitations. For example, a metal mesh HE has poor compatibility with the resin and increases the joint weight [134,135]. A carbon fiber HE is limited by factors such as the connection interface shape, the electrical connection quality with the electrode [69,70], and low robustness of joint quality when a large number of fibers are used [134,136]. Therefore, researchers have developed new types of HEs such as carbon nanotubes (CNTs) and carbon black, which effectively avoid the above problems (Table 5). Russello et al. [134] used the tensile-winding method to make the fully oriented CNT array into a thick aligned CNT network, and the joint strength can reach 96% of the base material. Cao et al. [136] added two layers of staggered multi-walled CNTs to the edge of the CF heating element, and the edge temperature increased from 70~80 °C to 160~180 °C, which effectively reduced the temperature difference between the center and the edge. The joint LSS increased by 153.6% to 62.9 MPa.

#### 3.2.4. Resin Insulations

Adding an insulating layer between the base material and HE can effectively improve the joint electrical insulation and the temperature distribution uniformity, and ultimately improve the joint performance. However, researchers have found that different types of insulation layers have different effects on the joint performance.

Ageorges et al. [99] found that the GF/PEI film has a better electrical insulation effect than pure PEI film, and the interface temperature distribution of a CF/PEI joint and GF/PEI joint is more uniform. Cui et al. [86] found that the addition of disordered fibers to the film can produce synergistic strengthening of the interface with the SS mesh, thus strengthening the interface at different scales, and the failure mode is also changed from interlayer failure to SS mesh and fiber tearing. In addition, the film mechanical properties affect the enhancement effect. Among them, CF/PEI film has the best performance and the best enhancement effect on SS mesh, followed by GF/PEI film, AF/PEI film, and pure PEI film is the worst. Sun et al. [129] found that after PEI films on both sides of CF fabric were modified by hexagonal boron nitride, the bonding with CF heating elements was stronger, the size and number of interface holes were reduced, and the joint LSS was increased by 31.4%. Xiong et al. [138] found that compared with pure PEI film, when a CFF/PEI composite film is selected, it not only accelerates the heat transfer in the welding area, but also has its own heat generation, and can effectively strengthen the interface. The GF/PEI joint LSS was increased by 16.5%, and the welding time was shortened by 25%.

### 3.3. Other Factors

In addition to the above process factors, the fiber orientation [65], the moisture content of the base material [33], the cooling rate, and other factors also affect the FRTP joint strength.

#### 3.3.1. Fiber Orientation

The orientation and type of the reinforcing fiber in the base material, heating element, and insulating layer all affect the final joint performance. When the fiber orientation on the surface of the base material is perpendicular to the load direction, the joint LSS is reduced by 13~20% [33]. When there are more warp fibers on the surface, the joint is fiber–matrix degummed. When there are more weft fibers, it is mainly the tearing of the laminate. The higher the waviness of the weft fiber, the more tortuous the fracture path, and the higher the joint LSS [32,139]. Since the fiber sizing agent affects the adhesion between the fiber and the matrix, the joint LSS using the chromium methacrylate sizing agent is 238% of the aminosilane sizing agent. Compared with the CF/PEEK joints of quasi-isotropic fiber, the joint current shunt of unidirectional fiber is more obvious [71].

When the fiber orientation in the HE is perpendicular to the load direction, the LSS of CF/PEEK [65], GF/PP [140], GF/PA6 [140], and GF/PEI [33] joints is lower. Compared with the joint using a unidirectional carbon fiber HE, the LSS and G_Ic_ of the joint with a woven carbon fiber HE are increased by 69% and 179%, respectively, and the process window is also effectively broadened [95]. Compared with unidirectional insulating layers, the use of woven insulating layers results in a 17% and 69% increase in the LSS and G_Ic_ for CF/PEI joints, respectively.

#### 3.3.2. Base Material Water Content

The water absorption of different thermoplastic matrices is different. The processing pores induced by water in the base material during welding will change the displacement value, porosity, and joint failure mode [33]. Shi et al. [74] found that when the welding pressure is less than 1.5 MPa, the residual moisture in the GF/PEI laminate is the main cause of the volatile pores, and drying before welding can effectively reduce the pores in the joint. Since then, they [106] found that when the welding power was 80 kW/m^2^, the time for the completely dry base material and the base material with a water content of 0.1% to reach the threshold value of 25.6 MPa was 40~100 s and 30~75 s, respectively. The analysis shows that processing pores improves the thermal insulation performance of the base material and accelerates the heating process of the welding interface.

#### 3.3.3. Cooling Rate

The cooling rate during resistance welding affects the crystallinity of the thermoplastic resin matrix, which in turn affects the joint properties. In general, lowering the cooling rate can prolong the nucleation and crystallization time of the resin, increase the joint crystallinity, which is beneficial to the tensile strength, hardness, and heat resistance of the joints, but is detrimental to the elastic properties, elongation at break, and impact the strength of the joints [106,130]. However, when the cooling rate is too slow, large crystals appear in the thermoplastic matrix, reducing the joint properties. When the cooling rate is too fast, a large amount of thermoplastic resin fails to nucleate and grows, resulting in lower crystallinity and poorer joint properties.

Li et al. [106] controlled the cooling rate after welding by adjusting the current, thereby regulating the crystallinity and crystal morphology. Reducing the cooling rate can increase the crystallinity of CF/PEEK joints, thereby improving the LSS and fatigue properties of the joints. When the cooling rate is 65 °C/min, the PEEK crystal grows in three dimensions, the joint LSS reaches a stable value of 46.2 MPa, and the fatigue performance is also improved. Wang et al. [130] found that when the cooling rate is less than 20 °C/min, the LSS of CF/PPS joints increases with the increase in the welding cooling rate. When the welding cooling rate is 20 °C/min, the joint LSS reaches a peak value of 7.2 MPa.

#### 3.3.4. Ultrasonic-Assisted Effect

**Figure 9:**
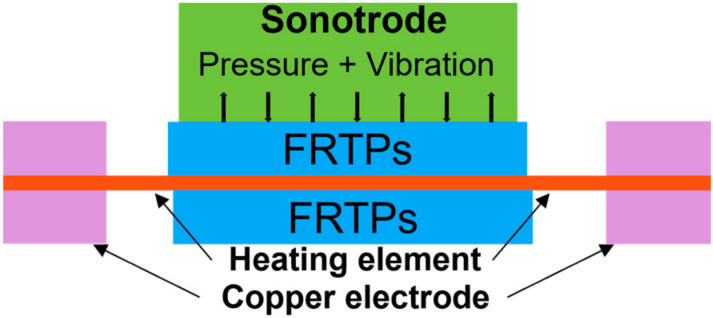
Schematic diagram of ultrasound-assisted resistance welding setup [141].

To improve the quality of FRTP resistance-welded joints, researchers have proposed an ultrasonic-assisted resistance welding method (Figure 9). Wang [119] found that ultrasonic action can improve the joint crystallinity, promote the flow of resin at the interface, and ultimately reduce and eliminate pores. When a 100-mesh SS mesh was used as the heating element, the GF/PPS joint LSS increased by 20~30% after an ultrasonic treatment of 2 s. However, when the ultrasonic treatment was too long, excessive heat input can cause resin degradation, leading to a reduction in the joint strength. Ma et al. [141] also found that ultrasonic action reduced the temperature difference between the center and the edge of the welding interface. With an ultrasonic treatment time of 7.5 s, the GF/PPS joint LSS increased by 19%, and the welding time was reduced by 40%.

#### 3.3.5. Environmental Factors

Researchers also studied the effects of contaminants such as moisture and oil on the joint performance. Overall, the impact of pollutants is related to the environmental resistance of materials. Yerra et al. [142] found that the oil residue on the interface greatly weakened the joint strength of the CF/PA66 interface, which was 74.7% and 68.8% of the original joint at 5 μL and 8 μL, respectively. The amount of pollution has little effect on the joint strength. However, since the literature on this topic is very limited, this phenomenon needs further validation.

The effect of moisture on joint properties is related to the hygroscopicity of the material. Yerra et al. [142] found that water diffuses into the CF/PA66 interface and reduces the joint strength by hydrolyzing chemical bonds. When there was less moisture (6 μL), the joint LSS decreased by only 4%. But when the water content was higher (9 μL), the LSS decreased by about 29.9%. Rohart et al. [123] found that an aminopropyltriethoxysilane (APTES) coating can not only effectively improve the adhesion between an SS mesh and PPS matrix, but also effectively prevent moisture absorption at the joint interface. After APTES treatment, the joint LSS increased by 32%. Xiao et al. [127] found that due to PEEK’s poor moisture absorption and strong environmental resistance, CF/PEEK joints did not significantly decrease their LSS after 360 h in an environment with a temperature of 85 ± 5 °C and a humidity of 90%.

#### 3.3.6. Service Temperature

The service temperature has little effect on the joint strength but affects the internal performance of the base material by affecting the residual thermal stress inside the base material and the static friction between the fiber and the matrix. Koutras et al. [143] found that at −50~150 °C, the GF/PPS joint LSS gradually decreased with the increase in the temperature, but the interface connection was good, which was the failure between the GF and matrix. At −50 °C and 150 °C, the joint LSS was 128% and 65% of the LSS at room temperature, respectively. At 50~90 °C, the joint LSS is basically unchanged. Rohart et al. [144] also found that the CF/PPS joint LSS decreased linearly with the increasing temperature in the range of 21 to 150 °C. The linker LSS at 82 °C and 150 °C was 74% and 39% of that at room temperature, respectively. They [145] chose the SS mesh after APTES treatment as the HE of a CF/PPS joint and found that after 1000 freeze–thaw cycles at −40~80 °C, although the fiber–matrix interface and the SS mesh/matrix interface are affected by environmental conditions, the joint LSS will not be significantly reduced and is still higher than before the treatment.

## 4. Failure Analysis of Resistance-Welded Joints

To accurately evaluate the performance of FRTP resistance-welded joints, researchers have adopted the lap shear (LS) strength test, double-cantilever beam (DCB) test, three-point/four-point bending test, mixed-mode bending test, and other test methods, among which the LS and DCB tests are the most common testing methods [40,109,146].

**Figure 10:**
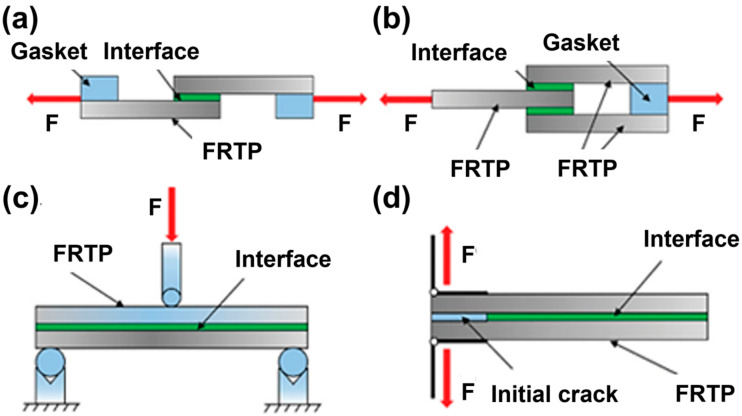
Schematic diagram of (**a**) single-lap shear strength (SLSS) test; (**b**) double-lap shear strength (DLSS) test; (**c**) interlaminar shear strength (ILSS) test; (**d**) double-cantilever beam (DCB) test.

In the LS test, the single-lap shear strength (SLSS) test (Figure 10a) is the most commonly used test. The double-lap shear strength (DLSS) test (Figure 10b) significantly reduces the interference of out-of-plane stress by minimizing the load eccentricity, and the measurement results are more accurate [147]. The interlaminar shear strength (ILSS) test (Figure 10c) is often used to characterize the interaction and failure of the laminates within the joint [109]. The DCB test (Figure 10d) was originally used to evaluate the performance of bonded joints and was supplemented by the LS test to support the evaluation of the fracture toughness of the joints. The results are usually expressed as the interlaminar fracture toughness G_IC_ [123].

**Figure 11:**
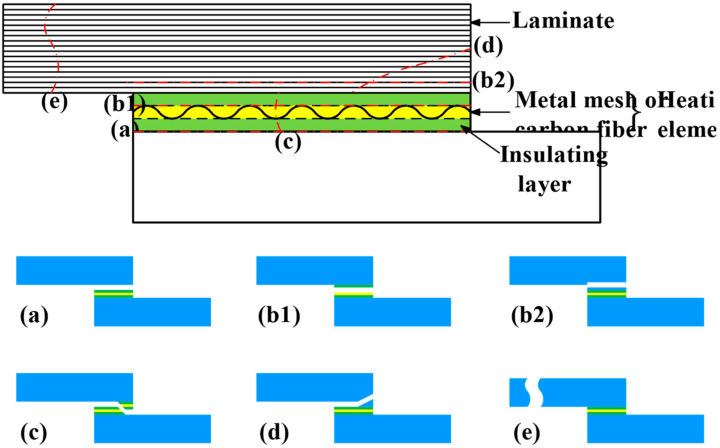
Failure modes classified by macroscopic fracture location [56,73,86]: (**a**) interfacial failure; (**b1**) interlaminar failure in the HE; (**b2**) interlaminar failure in the base material; (**c**) HE tearing; (**d**) intralaminar failure; (**e**) base material failure.

Due to the presence of an HE in a resistance-welded joint, its fracture mode is more complex. According to the macroscopic fracture location, the failure modes of FRTP lap joints can be divided into interfacial failure, interlaminar failure (interlayer failure [62], interlayer shear failure [59]), base material failure [75], HE tearing, and intralaminar failure, as shown in Figure 11. Fracture at the contact interface between the HE and FRTPs, which does not involve the damage of the HE and the base material, is called interfacial failure (Figure 11a) [59]. It usually occurs in joints with insufficient resin filling, poor wettability and high porosity at the interface, and its overall performance is poor. The fracture of the joint occurs through the interface of the SS mesh layer and insulating layer in the HE (Figure 11b1) or through the interface between adjacent laminates in the base material (Figure 11b2), which is called interlaminate failure. If the joint fractures through the thickness direction of the HE, the HE is torn off. In this case, the failure mode is called HE tearing, as shown in Figure 11c. Fraction that occurs through the thickness direction of the laminated base material is called intralaminar failure, as shown in Figure 11d. The fracture of FRTP base material far away from the welding zone is called base material failure (Figure 11e), which usually occurs in base material with a joint strength greater than the FRTP strength.

According to the microscopic fracture mechanism, the failure modes of FRTP lap joints can be divided into cohesive failure, adhesive failure, fiber–matrix debonding, carbon fiber fracture, etc. A cohesive failure usually occurs within the resin. Interfacial failure can be considered a cohesive failure because the insulating layer and base material have the same resin matrix, and fracture occurs within the same resin. If a metal mesh is used in an HE, then the interlayer failure between the metal mesh and the insulating layer is considered as adhesive failure.

### 4.1. Failure Analysis under Static Load

**Figure 12:**
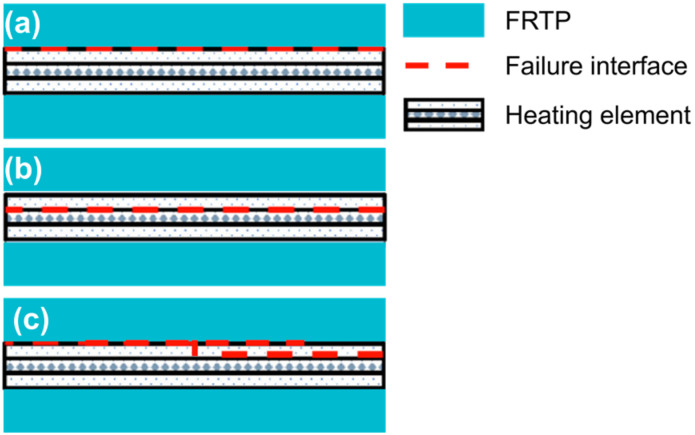
Surface integrity and microstructure of typical failure modes [56]: (**a**) interfacial failure; (**b**) interlayer failure; (**c**) mixture failure.

Hou et al. selected CF [61] and SS mesh [81] heating elements and found that the macroscopic fracture mode of CF fabric/PEI resistance-welded joints under a static-load LSS test is interfacial failure, while the microscopic fracture mechanism is cohesive failure. Yu et al. [78] found that after an oxidized treatment of a brass mesh HE, the macroscopic fracture mode of the CF/PPS joint changed from interface damage to interlayer structural damage, and the microscopic fracture mode was carbon fiber fracture. Barbosa et al. [113,120] found that when the shear tip appeared in the cohesive matrix fracture zone and the fiber–matrix interface of the CF/PPS and GF/PPS joints, the interface bonding was good, and the joint was interlaminar failure. The joint interface with a low LSS is only mechanically anchored, and the resin matrix fails to effectively impregnate the heating element, which is manifested as interfacial failure. Du et al. [56] found that after a three-point bending test of a GF/PP joint, the fracture position of the joint is interfacial fracture (Figure 12a) and interlaminar fracture (Figure 12b), and the microscopic fracture mechanism is cohesive failure (Figure 12a), adhesive failure (Figure 12b), and mixture failure (Figure 12c).

**Figure 13:**
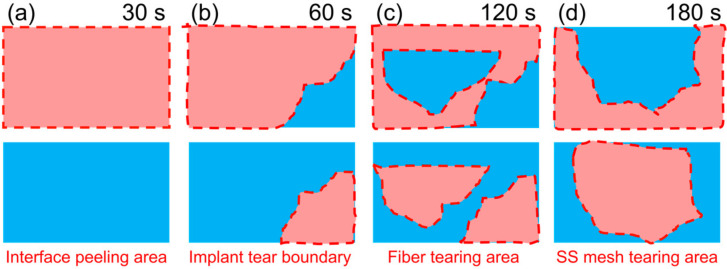
Bonding state of resin and SS mesh and failure mode under different welding times [86]: (**a**) interlayer failure; (**b**) SS mesh linear tearing; (**c**) SS mesh and fibers tear simultaneously; (**d**) SS mesh circular tearing.

Cui et al. [86] found that the joint failure mode varied with the welding time: at a welding time of 30 s, the resin was only partially melted, resulting in a lower bonding strength with the SS mesh, and the joint exhibited interlaminar failure (Figure 13a). At a welding time of 60 s, the molten resin increased, and the bonding strength with the SS mesh was greater. However, the interface edge temperature was still higher than the center temperature, and the joint was linear tearing (Figure 13b). At a welding time of 120 s, the resin was fully melted, the bonding strength with the SS mesh was the largest, and the joint quality was the best, characterized by simultaneous tearing of the SS mesh and fibers (Figure 13c). When the welding time was too long (180 s), the resin at the edge was overheated and degraded, weakening the bonding strength with the SS mesh, showing annular tearing (Figure 13d).

When the carbon fiber HE is selected, enhancing the adhesion between the fibers and the matrix can effectively improve the joint performance. Ageorges et al. [96] found that when the welding time is too short or the welding power is low, the adhesion between the fiber and the matrix is poor, the joint is mainly HE interfacial failure, and the fracture mechanism is adhesive failure. When the welding time is too long or the welding power is large, the FRTP deconsolidates and undergoes interfacial tilting deformation, resulting in specimen failure. With appropriate process parameters, the bonding between the fibers and the matrix is excellent, and joint failure is mainly due to the heating element failure or interlayer failure.

### 4.2. Failure Analysis under Fatigue Load

In addition to static load analysis, it is also important to analyze the fatigue properties of resistance-welded joints of FRTPs under a dynamic load. Researchers have found that ensuring good wettability of the reinforcing fibers to the matrix, i.e., by adjusting the welding process to make the molten polymer flow and spread better in the reinforcing fiber, helps to improve the joint fatigue strength.

Dubé et al. [105] selected SS mesh and found that CF/PEI, CF/PEKK and GF/PEI resistance-welded joints obtained infinite fatigue life at 25%, 25%, and 20% of static LSS, respectively, and the fatigue performance was better than that of bonded joints. The joint fatigue performance is closely related to the interfacial bonding, whereas the peeling stress makes the SS mesh and the edge of the matrix debond, and the crack expands in the SS mesh thickness direction, which ultimately leads to the tearing of the SS mesh. Therefore, the peeling stress has an important effect on the joint fatigue performance.

Later, they [147] found that the DLSs of CF/PEEK, CF/PEKK, CF/PEI, and GF/PEI were 53 MPa, 49 MPa, 45 MPa, and 34 MPa, respectively, which were basically the same as their respective LSSs. When the load level is 20%~30% of the static LSS, the above joints obtain infinite fatigue life. The main fracture locations for CF/PEEK, CF/PEKK, and CF/PEI joints were the SS mesh, SS mesh and substrate, and substrate, respectively. In addition, the fracture mode of all the above joints under static load and fatigue conditions were interlaminar failure. The GF/PEI joints were interlaminar failure only when the load was small, and the rest of the cases were base material failure. The above failure modes reflect the different degree of adhesion between SS mesh and the substrate. It is further found that PEKK and PEEK are semi-crystalline polymers, and the adhesion strength with SS mesh and reinforced fiber is greater than that of amorphous PEI.

Iqbal et al. [148] compared riveted, bonded, resistance-welded, and hybrid connections of GF/PP and found that resistance-welded joints had the greatest static load strength and the least change in joint modulus due to dynamic tensile loading at almost the same number of cycles to failure (39,674) as the mechanically connected joints (40,100) but at more than six times the load level.

## 5. Numerical Simulations of FRTP Resistance Welding

During FRTP resistance welding, the temperature distribution at the weld interface has an important effect on the joint performance. Researchers have adopted the analytical method [149], differential method [150], and finite element method (FEM) [66,83,93,118,127] to simulate the heat generation and heat transfer process during the resistance welding of FRTPs and the residual stresses in the workpiece after welding. According to the simulation content, this section will summarize thermal simulation and mechanical simulation, respectively.

### 5.1. Thermal Simulation

Table 6 summarizes the thermal simulation of FRTP resistance welding. The main software used includes ABAQUS 2020, COMSOL 5.3, and ANSYS 2020, and the geometric models include one-dimensional (1D), two-dimensional (2D), and three-dimensional (3D). The material models that can be used for resistance welding of FRTPs include anisotropic models [93], viscoelastic models [10,54], etc. The commonly used finite elements were 8-noded bricks (for ABAQUS) and PLANE55 (for ANSYS). The contact of the layers is mainly realized by thermal gap conductance for the heat exchange [151,152]. But most of the literature does not mention this information.

As early as 1989, Jakobsen et al. [93] found through simulation that the resistivity and heat loss of an HE would affect the optimum welding time and successfully predicted the edge effect. Xiao et al. [127] found that although the latent heat of crystallization during CF/PEEK resistance welding was neglected, it was basically the same as the actual measurement. The input energy, heating time, and the type of insulation layer will affect the accuracy of the model. Holmes et al. [151] found through simulation that the latent heat of crystallization of the substrate affects heat transfer and achieving good insulation reduces the minimum power for high-quality joining.

Ageorges et al. studied the joint properties in terms of joint thermal conductivity [152], densification state [153], and crystallinity [154] and determined the optimum process window. The process window was wider when braided CF was selected than when unidirectional CF was selected [153], and the time required to achieve close contact correlated well with the optimum welding time [96]. They [152] accurately predicted the thermal value of CF bundles by setting the thermal gap conductivity of the 3D model and obtained the relationship between the welding power and the t_m_ (melting time), t_g_ (glass transition time), and t_d_ (thermal degradation time) of the resin matrix. However, contact resistance and wire resistance were not considered in the model, and the simulated temperatures were slightly higher than the experimental measurements. They [83] also modeled impulse resistance welding (IRW) and found that when the HE was embedded in the electrode, the temperature distribution at the interface was more uniform and the welding time was shorter.

Researchers have also used thermal simulation to guide the welding process. Ageorges et al. [83] found that an increase in the welding power resulted in a significant decrease in the process window and a decrease in process flexibility and repeatability. Talbot et al. [66] found that although an increase in power reduces the weld time, it also reduces the process window and the substrate at the edges is more susceptible to overheating degradation. They [68] also found that controlling the heat transfer between the clamping distance and the substrate length was effective in improving the interfacial uniformity. Gouin et al. [112] predicted the temperature–time profile by modeling and used it to guide the welding process. Shi et al. [155,156] optimized the clamping distance based on a longitudinal heat transfer model and optimized the overlapping edge distance based on a transverse model. They also established the process window by simulating the polymer crystallization kinetics with complete curing and a degradation rate of less than 0.1% as the conditions. Brassard et al. [70] designed a conductive PEI/MWCNT as the HE and predicted the processing window based on thermal simulation. Zhang et al. [157] provided strong evidence for the initiation mechanism of the internal defects by simulation, and also revealed different PEEK melting patterns at different regions of the weld. De et al. [158] guided the selection of the process parameters by the temperature simulation of CF/PEEK resistance welding.

The above study considered the effect of the temperature on material properties, HE resistance, and the latent heat of crystallization of the resin matrix. Among them, the effect of the latent heat of crystallization on the overall heat transfer is small due to the limited amount of resin matrix in the resistance welding process. The combination of theoretical simulation and experimental verification can deepen the understanding of heat generation, heat transfer mechanism, interface temperature distribution, and its influence on the comprehensive performance of joints during FRTP resistance welding, which is of great significance for the optimization of subsequent welding process parameters.

### 5.2. Mechanical Simulation

The mechanical simulation of FRTP resistance welding is mainly used to study the residual stress and failure of the workpieces after welding. At present, the software selected by researchers for mechanical simulation is ABAQUS, and other simulation details are shown in Table 7.

Nagel et al. [159] found through simulation that the magnitude and distribution of stresses were related to the fiber direction of the substrate, HE, and insulation. It is shown that the stresses along the fiber direction are moderate, including the tensile and compressive stresses. However, the stresses perpendicular to the fiber direction are just larger tensile stresses. Transverse stresses were present near the splice region, which could lead to microcracks in the substrate. Shi et al. [118] combined the FEM with the digital image measurement (DIC) and found that the highest stresses were found at the lap edges, while the SS mesh edges were less stressed. The choice of rounded corners significantly reduced the strain concentration at the lap edges and increased the joint LSS by 86.6%.

Some researchers have also modeled the failure of FRTP resistance welding. Guo [46] found that without fiber bridging, the interlaminar fracture toughness was mainly determined by the interfacial properties of the fiber/substrate, and the ply thickness did not have much effect on the interlaminar fracture toughness of the DCB specimens. Luo et al. [160] used ABAQUS finite element software to establish a 2D plane strain model of a CF/PEEK single-lap resistance-welded joint to simulate the tensile–shear process. In this model, the element of the base material was a CPS4R type, the element of the weld was a COH2D4 type, and the boundary conditions were consistent with the actual tensile–shear test. The cohesion models of exponential, bilinear, and trapezoidal types were given to the weld through the user subroutine, the damage initiation criterion adopted the secondary nominal stress criterion, and the damage evolution criterion adopted the two-dimensional B-K criterion. It was found that the three models yielded the same stiffness, which increased linearly in the initial stage, in general agreement with the tests. The errors between the LSS predicted by the three models and the joint experimental mean value were 3.7%, 12.8%, and 7.4%, respectively.

Taken together, the mechanical simulation of FRTP resistance welding mainly focuses on the prediction of post-weld residual stresses and various types of strength of the joints. And with the improvement in the scientificity and accuracy of the selected model, the prediction results are becoming more and more accurate.

## 6. Process Control and Quality Inspection

Since the energy of FRTP resistance welding comes from the Joule heat generated by the HE after energization and is closely related to the pressure effect during cooling and solidification, monitoring the information such as the heat distribution, resistance change, and displacement change at the interface is crucial for process control and quality inspection [163]. In terms of quality inspection, researchers mainly use ultrasonic testing [108,122,130] (UT), acoustic emission testing (AE) [114], laser sensor monitoring [89], and radiographic testing (RT) for non-destructive testing.

Temperature can be directly recorded by an infrared camera, thermal imager, thermocouple, etc., or indirectly estimated based on the HE resistance and welding current [164]. Tanabe et al. [165] recorded the temperature change during CF/PPS resistance welding by thermal imaging and used image analysis to measure the welding area ratio and monitor the power-on situation. It was found that the lap area could be expanded by only increasing the lap width and welding current. However, due to the edge effect, only increasing the lap length has a poor effect on expanding the lap area. Zammar et al. [166] designed a fuzzy logic controller based on the real-time temperature for continuous resistance welding. The welding voltage and welding speed were closed-loop controlled by temperature feedback, and the temperature was successfully controlled within 10%. The accuracy of the indirect estimation of the interface temperature by the welding current is better, but attention should be paid to the robustness and accuracy of the temperature feedback. Overall, closed-loop control is more effective than open-loop control.

Since the resistance of the SS mesh is approximately linear with its heat production, and the heat production is closely related to the temperature.Villegas et al. [164] proposed converting the target interface temperature into the target resistance value of the SS mesh for program control. However, it was found that the relationship between the SS mesh resistance and temperature obtained by different test methods was also different, which may produce a large control deviation in the actual operation. Okayasu et al. [167] controlled the resistance, and consequently the interfacial heating temperature, by regulating the amount of carbon fibers exposed in CF/PPS. It was found that when a greater amount of carbon fibers were exposed, the resistance was lower, resulting in less heat generation and a limited heating area.

Since the extrusion flow and void elimination of the resin during welding are closely related to the displacement change [10], the welding process can also be monitored by measuring the vertical displacement at the interface. Evenos et al. [60] controlled the welding process by measuring the pressure change during constant displacement resistance welding and combining the interface temperature measured by thermocouples. Ageorges [96] found that the decrease in the joint thickness during welding is related to the joint LSS, which provides a new idea for the on-line monitoring of FRTP resistance welding. By analyzing the welding displacement curve, Shi et al. [106] obtained the interface changes in the process of close contact and extrusion flow, which effectively shortened the time of formulating the process window.

## 7. Thermoplastic/Thermoset Composite Resistance Welding

**Figure 14:**
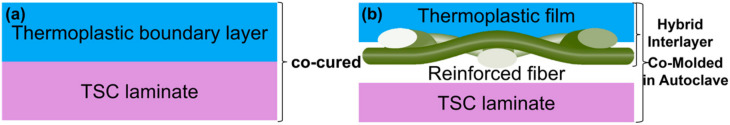
TSC surface thermal plasticization technology [169]: (**a**) co-curing of thermoplastic film; (**b**) mixed thermoplastic film.

Although the application of thermoplastics (TPCs) in aerospace and other fields has increased year by year, thermoset plastic (TSC) is still widely used in large and important structures. Therefore, it is also important to achieve low-cost and efficient joining between TPC and TSC. However, the welding of TPC and TSC has two major problems: mutual bonding and TSC thermal degradation [168]. To this end, researchers usually employed thermoplastic film co-curing technology (Figure 14a) and thermoplastic mixed interlayer technology (Figure 14b) [4,8] to add a thermoplastic resin layer at the interface to improve the weldability and adhesion of the TSC surface [169]. It avoids TSC degradation due to overheating [10], and finally realizes the effective joining of TPC to TSC [4]. Jacaruso et al. [170] proposed that the resistance welding of TPC-TSC can be realized by relying only on the bonding characteristics of TPC without using a thermoplastic resin layer.

### 7.1. Co-Curing of Thermoplastic Films

This method is based on the adhesion between the thermoplastic film and the TSC resin layer, and the surface thermoplasticization of TSC is realized by the co-curing effect of an autoclave or hot press. The process is like diffusion bonding. Jacaruso et al. [171], Hou et al. [172], Lionetto et al. [173], and Quan et al. [174] have prepared thermoplastic films as the intermediate layer of TSC-TSC welding by co-curing technology. Xie [175] combines thermoplastic binder co-curing and blending layers and uses three different thermoplastic binders to achieve high-quality and efficient joining of TSC. Based on this, researchers have proposed using a TP layer compatible with the TSC resin matrix and co-curing it with the TSC base material [176,177]. It can also be combined with TP by selecting a third polymer that is compatible with both TSC and TPC [8]. After curing, the TP layer and TSC form a mutually penetrating network structure. Overall, the process is simple, and the chemical compatibility of the TP film and TSC resin matrix is high.

To enhance the interfacial bonding strength between a TP film and TSC, Jacaruso et al. [178,179] used plasma, corona, ultraviolet treatment, and other methods to make the surface of a TP film with a high chemical inertness rich in epoxy groups, and then co-cured it with epoxy. However, this process accelerated the thermal degradation of the resin. Cui et al. [180] also found that the co-curing of a modified TP interlayer can form a stable semi-interpenetrating polymer network (S-IPN) in the interface layer, which can realize the reliable joining between CF/Epoxy and GF/PEI.

### 7.2. Thermoplastic Hybrid Interlayers

Jacaruso et al. [171] proposed a thermoplastic hybrid sandwich TSC-TSC joint. In this method, TP polymer is impregnated on one side of the roving fiber cloth, and then TSC is cured, allowing TS resin to flow in the mixed interlayer, and relying on the braided fiber cloth to promote the mechanical interlocking of TS and TP [178]. This method can join all TSC and TPC and has a wider application range than thermoplastic film co-curing [176]. However, it should be ensured that the TP polymer has a high viscosity during curing, so as to ensure the mutual penetration of the TP through thickness.

**Figure 15:**
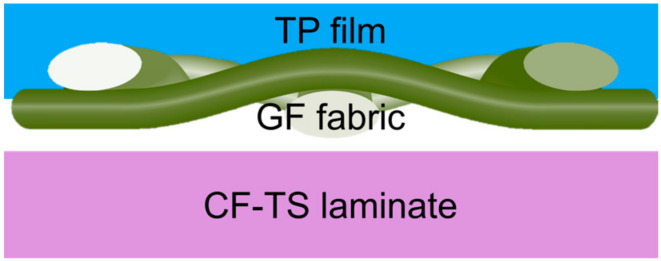
Schematic of a hybrid interlayer [181].

Ageorges et al. [181] effectively enhanced the CF-PEI/CF-EP joint strength by impregnating PEI and EP on both sides of a GF fabric. The structure of this hybrid interlater is shown in Figure 15. They also constructed a process window based on the minimum LSS of 15 MPa, but the established thermal degradation kinetic model overestimated the joint’s actual degradation, and still had limitations when applied to the case of a fast heating rate or short residence time, and the correlation between the predicted joint thermal degradation and the actual performance was poor.

## 8. Conclusions and Prospects

In this paper, the research status of FRTP resistance welding is reviewed from six aspects: the basic process of FRTP resistance welding, the influencing factors of joint performance, failure analysis, numerical simulation, process control and quality monitoring, and TSC/TPC resistance welding. The main conclusions and prospects are given as follows:

- Statistical studies show that the most important factor that influences the resistance-welded joint performance is the heating element, followed by the welding current/power, welding time, and welding pressure.
- The heating element not only affects the heat production and heat distribution, but also affects the stress distribution in the joint and the bonding of the interface. At present, surface modification, optimization design of the heating element, and the resin insulation layer are mainly used to improve the bonding between the HE and the base material, to improve the uniformity of the heat distribution and stress distribution at the interface, and to weaken or eliminate interface defects, the edge effect, and current shunt. However, the selection of the HE is still lacking theoretical guidance, and further research needs to be conducted.
- The properties of FRTP resistance-welded joints are also affected by non-technical factors such as the matrix type, fiber size, and orientation, the water content of the base material, and service environment conditions. However, systemic research on these non-technical factors is still insufficient.
- For the thermal simulation in FRTP resistance welding, the commonly used finite elements were 8-noded bricks (for ABAQUS) and PLANE55 (for ANSYS). The contact of the layers is mainly realized by thermal gap conductance for the heat exchange. Thermal simulation is mainly used to predict the temperature of the joint interface or heating element for further adjustment or selection of the process. However, the depth and breadth of thermal simulation is still limited. There still exists large research space in this field.
- For the mechanical simulation in FRTP resistance welding, the cohesion models of exponential, bilinear, and trapezoidal types, the damage initiation criterion of the secondary nominal stress criterion, and the damage evolution criterion of the two-dimensional B-K criterion were validated in predicting the mechanical performance.
- Monitoring process information such as the temperature distribution, resistance changes, displacement changes, etc., is essential for process control and quality inspection. Combining direct or indirect temperature measurement methods with closed-loop control can effectively improve efficiency and stability. Establishing a closed-loop control system for FRTP resistance welding has strong development prospects.
- Due to the large difference in the properties of TSC and TPC, the addition of a thermoplastic layer at the interface is crucial for achieving a reliable resistance-welded TPC/TSC joint. Co-curing a thermoplastic film or using a mixed thermoplastic interlayer are the two main technologies to add the thermoplastic layer. However, the material combination used for TPC/TSC dissimilar welding is very limited and needs more validation.

## Figures and Tables

**Table 1:** Effect of each process parameter of FRTP resistance welding.

Ref.	Materials	HE	Methodology	Contribution
[77]	CF/PLA	Polypyrrole, woven CF with SS mesh	ANOVA	(1) HE Type: Heating Time: Area Covered by HE = 88.5%: 6.6%: 1.2% (Tensile Properties) (2) HE Type: Heating Time: Area Covered by HE = 90.4%: 4.8%: 1.4% (Compressive Properties)
[59]	CF/PPS	CF/PPS blended fabric	Taguchi method, ANOVA	Current: Time: Pressure = 83.37%: 9.55%: 6.02%
[78]	CF/PPS	Brass mesh	Response surface method, ANOVA, fitted curve analysis	Power density has the greatest effect on joint performance and is strongly correlated with interfacial temperature, with welding pressure having the least effect
[79]	GF/PAS	Fabric CF/PAS	Taguchi method, ANOVA	Power: Time: Pressure = 53%: 37%: 10%
[56]	GF/PP	SS mesh	Orthogonal method, response surface method	Current: Time: Pressure = 76.2%: 23.8%: 0.0088%
[73]	GF/PP	SS mesh	Taguchi method	Current: Time: Pressure = 75.113%: 20.406%:0.417%
[80]	GF/PP	304 SS mesh	Taguchi method, ANOVA	Current: Time: Pressure = 58.12%: 23.07%: 15.29%

**Table 2:** Summary of research on resistance welding of FRTPs using stainless steel mesh heating elements.

Ref.	Materials	Thickness (μm)	Open Gap width/Wire Diameter (μm/μm)	Insulating Layer	Welding Pressure (MPa)	Energy-Related Parameters	Welding Time (s)	Best Strength (MPa)
[29]	CF/PA66	80	47/40	50 μm PA66 film	1~2.5	141.3 kW/m^2^	140	LSS: 24.1
[84]	CF/PEEK	80	-/80	PEEK film	-	526 °C/min to 450 °C	48.6	LSS: 49.01
[66]	CF/PEEK	80	90/40	127 μm PEEK film	1.0	6.0 V to 440 °C	-	LSS: 47.4
[104]	CF/PEEK	100	45/50	80 μm PEI film	0.4	15 A	6	LSS: 21.57
[88]	CF/PEEK	50	28/25	80 μm PEI film	0.3~0.5	15 A	6~8	LSS: 18.0
[67]	CF/PEEK	80	90/40	127 μm PEEK film	1.0	9.0 V to 440 °C	60	ILSS: 88.5
[67]	CF/PEEK	80	90/40	127 μm PEEK film	1.0	3 V/min to 440 °C	210	ILSS: 88.9
[105]	CF/PEEK	80	89/40	PEEK film	1.0	2 V, 10 V/min to 440 °C	-	LSS: 53.0
[106]	CF/PEEK	90	75/45	50 μm PEEK film	1.0	20 A	300	LSS: 36.09
[46]	CF/PEEK	140	150/70	PEEK film	0.8	at 360 °C	600	LSS: 11.04
[103]	CF/PEEK	80	87/40	60 μm PEEK film	1.0	66 kW/m^2^	225	LSS: 35.1
[107]	CF/PEEK	120	100/60	80 μm PEEK film	0.75	13 V	100	LSS: 17.26
[108]	CF/PEEK	100	74/50	80 μm PEEK film	1.0	45 A	30	LSS: 45.1
[105]	CF/PEI	80	90/40	PEI film	1.0	2 V, 9 V/min to 400 °C	-	LSS: 47.0
[105]	CF/PEI	80	89/40	PEI film	1.0	2 V, 10 V/min to 390 °C	-	LSS: 45.0
[109]	CF/PEI	60	55/30	-	0.7	50 A	80	ILSS: 63.7
[105]	CF/PEKK	80	90/40	PEKK film	1.0	2 V, 9 V/min to 420 °C	-	LSS: 52.0
[105]	CF/PEKK	80	89/40	PEKK film	1.0	2 V, 10 V/min to 410 °C	-	LSS: 49.0
[77]	CF/PLA	180	150/90	PLA film	0.1	AC 2 A	60	TS: 0.99 CS: 4.14
[110]	CF/PPS	80	90/40	PPS film	0.1	2 V, 0.27 V/min to 325 °C	60	LSS: 23.3
[111]	CF/PPS	116	74/58	PPS film	0.7	33.5 A	175	LSS: 17.13
[112]	CF/PPS	80	89/40	70 μm PPS film	0.5	130 kW/m^2^	55	LSS: 31.7
[113]	CF/PPS	-	200/-	-	0.7	33.5 A	175	LSS: 17.1 ± 1.4
[114]	CF/PPS	-	-	PPS film	0.8	at 360 °C	600	LSS: 8.26
[115]	CF/PPS	100	75/50	50 μm PPS film	2	42 A	10	LSS: 24.5
[105]	GF/PEI	80	90/40	PEI film	1.0	2 V, 9 V/min to 400 °C	-	LSS: 33.0
[105]	GF/PEI	80	89/40	PEI film	0.06	2 V, 10 V/min to 340 °C	-	DLS: 34.0
[33]	GF/PEI	80	90/40	127 μm PEI film	0.8	80 kW/m^2^	55	LSS: 32.04 ± 1.7
[116]	GF/PEI	200	160/100	200 μm PEI film	0.2	12 A	150	LSS: 25.1
[97]	GF/PET	80	90/40	127 μm PET film	1.38	32 A, 3114 kJ/m^2^	-	LSS: 25.61
[117]	GF/PP	460	1357/230	150 μm polyesters film	1.0	25 A	20	LSS: 23.0
[73]	GF/PP	1200	2575/600	-	10.8	30 A	30	LSS: 8.832
[56]	GF/PP	300	550/150	GF/PP prepreg	0.05	10 A	60	FS: 60 ± 0.12
[80]	GF/PP	460	1500/230	-	2.5	12.5 A	540	LSS: 12.186
[118]	GF/PPS	80	90/40	PPS film	0.8	80 kW/m^2^	60	LSS: 24.45
[119]	GF/PPS	300	500/150	200 μm PEEK film	0.8	39 A	14	LSS: 13.87
[119]	GF/PPS	160	175/80	200 μm PEEK film	0.8	32 A	14	LSS: 12.78
[120]	GF/PPS		~	-	0.7	30 A	300	LSS: 9.6

**Table 3:** Summary of FRTP resistance welding studies on surface modification of metal mesh heating element.

Ref.	Materials	HE	Treatment	Main Findings
[71]	CF/PEEK	SS	TiO_2_ coating	(1) Less power loss due to current shunt, more uniform interface temperature, shorter welding time. (2) A 20% increase in joint stiffness, shift from complex failure to delamination at heating element and coating.
[121]	CF/PEEK	SS	Sandblasting	IFSS increased by 1% to 28.3 MPa; LSS increased by 3% to 37.3 MPa.
[121]	CF/PEEK	SS	Aryl diazo grafting	IFSS increased by 22% to 34 MPa; LSS increased by 9% to 40 MPa.
[121]	CF/PEEK	SS	Silane grafting	IFSS increased by 36% to 38 MPa; LSS increased by 23% to 45 MPa.
[122]	CF/PEEK	SS	Electrostatically spun nanofiber membrane	(1) Provides resin-enriched areas, reduces voids, and enhances HE impregnation with resin and mechanical interlocking with the interface. (2) LSS increased by 230% to 36.98 MPa, and infinite fatigue life at 20% static LSS.
[108]	CF/PEEK	SS	Spinning plasma treatment	LSS increased by 14.63% to 51.7 MPa.
[123]	CF/PPS	SS	APTES silane treatment	LSS increased by 32% to 37.6 ± 2.2 MPa for ethanol solvent and pH = 11.
[115]	CF/PPS	SS	KH550 silane treatment	LSS increased by 27% to 31.1 MPa for optimum treatment.
[78]	CF/PPS	brass	350 °C oxidation treatment 30 min	(1) At 100-mesh, 124~128 kW/m2, 313~314 °C, 60 s, 1.04~1.2 MPa, the average LSS reached 13.58 MPa. (2) The surface roughness was larger, which enhanced the bonding with the resin, and the defects were significantly reduced, and the LSS was enhanced by 26.56%.
[124]	GF/PEI	SS	7 mol/L HCl chemical etching	(1) The LSS increases and then decreases with increasing etching time(te), and the joints change from interlayer debonding to interlayer tearing failure. (2) The LSS increased by 27.7% to 35.44 MPa at 30 min and 150 s for te and tw, respectively.
[116]	GF/PEI	SS	Ethanol flame growth of CNTs	(1) Capillary action of highly porous CNT coatings increases wettability of HE with PEI resins. (2) With the increase in treatment time, the LSS increased and then decreased, with a maximum of 39.2 MPa.
[125]	GF/PEI	SS	Reduction of graphene oxide (RGO) wrapping treatment	The dynamic contact angle (DCA) increased from 89.8° to 127.7° after 5 wraps, and the LSS increased by 54.14% to 41 MPa.

**Table 4:** Summary of FRTP resistance welding studies with selected carbon fiber heating elements.

Ref.	Materials	HE	Insulating Layer	Welding Pressure/MPa	Energy-Related Parameters	Welding Time/s	Best LSS/MPa
[126]	CF/PA6	Single-layer CF wire	PA6 film	1.0	0.6 kW heating to 260 ± 20 °C	30	21.6
[79]	CF/PAS	CF/PAS Prepreg	CF/PAS prepreg	1.5	50 kW/m^2^	50~180	6.6
[127]	CF/PEEK	CF/PEEK Prepreg	127 μm PEEK film	1.2	90~120 kW/m^2^	120	33.9 ± 2.3
[107]	CF/PEEK	Single-layer T700	80 μm PEEK film	0.75	27 V	105	21.14
[107]	CF/PEEK	Braided T700	80 μm PEEK film	0.75	27 V	90	15.54
[128]	CF/PEEK	UD * T700(PSI **)	PEEK film	0.75	27 V	120	28.1
[128]	CF/PEEK	Braided T700(PSI)	PEEK film	0.75	27 V	120	23.7
[61]	CF/PEI	Woven CF/PEI Prepreg	76 μm PEI film	0.3	118 kW/m^2^	20	31
[96]	CF/PEI	UD CF/PEI	PEI film	0.4	69 kW/m^2^	90	25
[96]	CF/PEI	Woven CF/PEI	PEI film	0.4	69 kW/m^2^	90	26
[96]	CF/PEI	Woven CF/PEI	PEI film	0.4	100 kW/m^2^	90	36
[129]	CF/PEI	Plain CF Fabrics	Hexagonal BN + CF/PEI	1.0	21 A	10	19.3
[77]	CF/PLA	Woven CF/PLA	PLA film	0.1	AC 2 A	60	1.32
[77]	CF/PLA	Woven CF/PLA	PLA film	0.1	AC 2 A	90	4.62 (compressed)
[130]	CF/PPS	Woven T300	PPS film	0.6	Heat to 390 °C, cool at 20 °C/min	-	9.13
[59]	CF/PPS	CF/PPS blended fabric	50 μm PPS film	1.5	12 A	1800	17.88
[76]	CF/PPS	CF/PPS blended fabric	-	1~1.5	80~170 kW/m^2^	300~1200	17.92
[96]	GF/PEI	Woven CF/PEI	PEI film	0.4	100 kW/m^2^	90	30
[87]	GF/PEI	CF	200 μm PEI film	0.2	10 A	60	26.3
[87]	GF/PEI	CF-CNT	200 μm PEI film	0.2	10 A	60	32.5
[131]	GF/PEI	GO *** + CF	100 μm PEI film	1.5	20 A	90	39.5

* UD: unidirectional. ** PSI: powder suspension impregnation. *** GO: graphite oxide.

**Table 5:** Summary of FRTP resistance welding research with new types of HEs.

Ref.	Materials	HE	Insulating Layer	Welding Pressure/MPa	Energy-Related Parameters	Welding Time/s	Best LSS/MPa
[69]	CF/PEEK	10% MWCNTs + PEI	-	1.0	350 kW/m^2^	120	19.6 ± 3.5
[70]	CF/PEEK	MWCNT	PEI film	1.0	350 kW/m^2^	120	24.9 ± 9.7
[134]	CF/PEEK	CNT-web	PEEK film	4.0	55 kW/m^2^	-	Tensile: 95 ± 3
[136]	CFRTPs	CF + 2-layer MWCNT sheet	Elium film	0.5	6.9 A, 9 kW/m^2^	1200	62.9
[135]	GF/PEKK	CNT mesh	PEKK film	0.05	80~90 kW/m^2^	50	30.0
[137]	PC	5%PANI, 8%CB, 87%ABS	-	0.3	40 V	240	25.4

**Table 6:** Summary of thermal simulation studies of resistance welding of FRTPs.

Ref.	Research Purpose	Software	Geometric Model	Element Type	Material	Relative Error
[93]	Prediction of melting time	-	2D model	-	CF/PEEK	-
[127]	Guiding process parameters	ANSYS	2D 1/4-symmetric model	-	CF/PEEK	<12%
[151]	Heat conduction	ABAQUS	1D and 2D model	DC2D4	CF/PEEK	<12%
[152]	Time to melt and time to cause thermal degradation	ABAQUS	3D 1/4-symmetric model	8-noded bricks	CF/PEEK, CF/PEI	<5%
[153]	Guiding process parameters	ABAQUS	3D 1/4-symmetric model	8-noded bricks	CF/PEEK, CF/PEI	-
[154]	Cooling rates	ABAQUS	3D 1/4- symmetric model	8-noded bricks	CF/PEEK, CF/PP	-
[99]	Interfacial temperature history	ABAQUS	3D 1/4-symmetric model	8-noded bricks	CF/PEI, GF/PEI	-
[96]	Time required for close contact and optimal welding time	ABAQUS	3D 1/4-symmetric model	8-noded bricks	CF/PEI, GF/PEI	-
[83]	Interfacial temperature distribution	ABAQUS	3D 1/4-symmetric model	8-noded bricks	CF/PEEK	<5%
[66]	Heat conduction	ANSYS	2D axisymmetric model	PLANE55	CF/PEEK	-
[66]	Z-direction heat conduction	ANSYS	3D axisymmetric model	SOLID70	CF/PEEK	5%
[155]	Interfacial temperature distribution	-	2D axisymmetric model	-	GF/PEI	<10%
[68]	Clamping distance	ANSYS	2D axisymmetric model	PLANE55	CF/PEEK	-
[68]	Clamping distance	ANSYS	3D axisymmetric model	PLANE55		-
[112]	Temperature–time curve	COMSOL	3D axisymmetric model	Tetrahedral solid elements	CF/PPS	<27%
[156]	Interfacial temperature distribution	COMSOL	3D axisymmetric model	-	GF/ PPS	
[70]	Processing window	COMSOL	3D axisymmetric model	Tetrahedral elements	CF/PEEK-PEI/MWCNT	<15%
[157]	Interfacial temperature distribution	-	3D model	8-node linear heat transfer hexahedron elements	CF/PEEK	-
[158]	Interfacial temperature distribution	ABAQUS	2D axisymmetric model	Planar deformable type	CF/PEEK	-

**Table 7:** Summary of mechanical simulation studies of resistance welding of FRTPs.

Ref.	Research Purpose	Geometric Model	Unit Type	Material	Error
[118]	Stress/strain analysis	2D	Planar shell elements	GF/PPS	<50%
[159]	Residual stresses	3D	Quadratic hexahedral, coupled temperature–displacement elements	CF/PEEK	-
[46]	Type I delamination of DCB specimens	3D	Cohesive element	CF/PEEK	<10%
[160]	Shear failure	2D	Laminates: CPS4R elements Welding interface: COH2D4	CF/PEEK	<13%
[161]	Rate-dependent experiments	2D viscoelastic interface model	Laminates and spacers: CPE4I Welding interface: COH2D4	CF/PEEK	-
[162]	Edgewise compression (EC) and 3-point bending (3PB) tests	3D	All units are C3D8R	CF/PP	EC: <13% 3PB: <27%

## Data Availability

Data are contained within the article.
